# Enteropathogenic *Escherichia coli* Uses NleA to Inhibit NLRP3 Inflammasome Activation

**DOI:** 10.1371/journal.ppat.1005121

**Published:** 2015-09-02

**Authors:** Hilo Yen, Nakaba Sugimoto, Toru Tobe

**Affiliations:** 1 Department of Biomedical Informatics, Graduate School of Medicine, Osaka University, Osaka, Japan; 2 Department of Microbiology and Immunology, Graduate School of Medicine, Osaka University, Osaka, Japan; University of Toronto, CANADA

## Abstract

Enteropathogenic and enterohemorrhagic *Escherichia coli* (EPEC and EHEC) are related strains capable of inducing severe gastrointestinal disease. For optimal infection, these pathogens actively modulate cellular functions through the deployment of effector proteins in a type three secretion system (T3SS)-dependent manner. In response to enteric pathogen invasion, the Nod-like receptor pyrin domain containing (NLRP) inflammasome has been increasingly recognized as an important cytoplasmic sensor against microbial infection by activating caspase-1 and releasing IL-1β. EPEC and EHEC are known to elicit inflammasome activation in macrophages and epithelial cells; however, whether the pathogens actively counteract such innate immune responses is unknown. Using a series of compound effector-gene deletion strains of EPEC, we screened and identified NleA, which could subdue host IL-1β secretion. It was found that the reduction is not because of blocked NF-κB activity; instead, the reduction results from inhibited caspase-1 activation by NleA. Immunostaining of human macrophage-like cells following infection revealed limited formation of inflammasome foci with constituents of total caspase-1, ASC and NLRP3 in the presence of NleA. Pulldown of PMA-induced differentiated THP-1 lysate with purified MBP-NleA reveals that NLRP3 is a target of NleA. The interaction was verified by an immunoprecipitation assay and direct interaction assay in which purified MBP-NleA and GST-NLRP3 were used. We further showed that the effector interacts with regions of NLRP3 containing the PYD and LRR domains. Additionally, NleA was found to associate with non-ubiquitinated and ubiquitinated NLRP3 and to interrupt de-ubiquitination of NLRP3, which is a required process for inflammasome activation. Cumulatively, our findings provide the first example of EPEC-mediated suppression of inflammasome activity in which NieA plays a novel role in controlling the host immune response through targeting of NLRP3.

## Author Summary

Enteropathogenic *Escherichia coli* (EPEC) and enterohemorrhagic *E*. *coli* (EHEC) cause severe intestinal dysfunction, including watery diarrhea or severe bloody diarrhea, and acute kidney failure (hemolytic uremic syndrome). Transmitted through ingestion of contaminated food, these pathogens colonize and disrupt the linings of intestinal epithelial cells. EPEC and EHEC interrupt many cellular functions, including the inflammation response, to increase their chances of proliferation and survival in the intestine. Upon detection of the invasion, epithelial cells and immune cells secrete inflammatory cytokines to further boost the immune response for efficient clearance of the pathogens. IL-1β is an important inflammatory cytokine, and its secretion is regulated by a multimeric protein complex, termed the inflammasome, in host cells. In this study, we discovered that EPEC injects a bacterial effector protein, NleA, to inhibit the secretion of IL-1β. Exploring the potential mechanisms, we found that NleA does so by directly associating with NLRP3 (Nod-Like Receptor 3), one of the three basic components of the inflammasome, and that the presence of NleA interrupts the de-ubiquitination of NLRP3, which is a prerequisite for the assembly of the inflammasome. As a result, NleA reduces the formation of the NLRP3 inflammasome and negatively regulates the secretion of IL-1β.

## Introduction

Enteropathogenic and Enterohemorrhagic *Escherichia coli* (EPEC and EHEC) are major causative agents of food poisoning worldwide [1]. EPEC causes infantile diarrhea, and EHEC causes bloody diarrhea and hemolytic uremic syndrome (HUS) in patients who ingest contaminated food [2]. These invading bacteria colonize the surface of the epithelial cells lining the intestinal tract and cause localized damage to the intestinal microvilli and rearrangement of host cytoskeletal proteins under the intimately attached bacterial colonies [3,4]. These characteristic histopathological lesions are referred to as “attaching and effacing lesions (A/E lesion)”, and EPEC/EHEC are known as “A/E pathogens” [5].

A/E lesion formation depends on a chromosomal region named the locus of enterocyte effacement (LEE), which is the key to EPEC/EHEC pathogenicity. LEE encodes the regulators, an adhesin (intimin), the chaperones, a translocator, the effector proteins and the type three secretion system (T3SS) components. The T3SS is a needle-like apparatus that is responsible for delivering the effector proteins into the cytosol of the host cells [6]. Effector proteins then modulate various aspects of cellular function and optimize bacterial infection. Based on the sequenced genomes of EPEC (E2348/69) and EHEC (O157:H7), more than 30 effector genes have been predicted, and at least 17 of these genes are found in both strains [7,8]. Several effector genes typically form clusters, and there are several of these clusters, known as pathogenicity islands, scattered in the genome; those effector genes located outside of the LEE are referred to as non-LEE-effectors. The functions of each effector protein are incompletely understood. Some effector proteins of the A/E pathogens have been shown to disrupt vital host cellular functions such as the cellular structures, cell death, proliferation and inflammatory responses [9]. Recently, the observation of pathogen-induced suppression of host inflammatory responses has led to the discoveries of multiple NF-κB (nuclear factorκ-light-chain-enhancer of activated B cells) pathway-inhibiting effector proteins, including NleB, NleC, NleE, NleH, EspL, and Tir [10].

Host cells are equipped with a variety of receptors on the membrane surface and within the cytoplasm to detect conserved bacteria-originated antigens as well as danger-associated molecular patterns (DAMPs) released from infected and damaged cells [11,12]. The ligand-receptor engagement elicits many downstream signaling events that result in cellular output of antimicrobial peptides, inflammatory cytokines, and chemokines required for further recruitment of innate and adaptive immune cells. Recently, the inflammasome, a multimeric protein complex consisting of Nod-like receptor (NRLs), ASC and caspase-1, was shown to be important in the maturation and secretion of interleukin (IL)-1β and related family members [12]. In particular, the NLRP3 inflammasome has been extensively studied and can be activated by a variety of stimuli including microbial infection [13–15].

Live non-pathogenic and pathogenic *Escherichia coli* are known to elicit the NLRP3 inflammasome [16–18], and such activation has been shown to be essential for clearance of *Citrobacter rodentium*, a mouse model pathogen of EPEC, in an animal infection model [19]. Because activation of the inflammasome leads to production of IL-1β and escalation of inflammation for enhanced pathogen clearance, counter measurements to this type of pathway appear to be vital for the survival of and successful infection by A/E pathogens. There have been no reports of A/E pathogen-mediated suppression of the inflammasome.

In this study, we identified NleA and NleE through screening of the effector genes of EPEC responsible for inhibiting IL-1β secretion. In contrast to NleE, NleA represses caspase-1 activation without affecting NF-κB activity. This inhibition is a result of reduced formation of inflammasomes containing NLRP3, ASC and total caspase-1/active caspase-1. Further examination revealed that NleA directly interacts with NLRP3 and affects the de-ubiquitination of NLRP3, which is known to control its activity.

## Results

### NleA inhibits host IL-1β secretion

**Fig 1:**
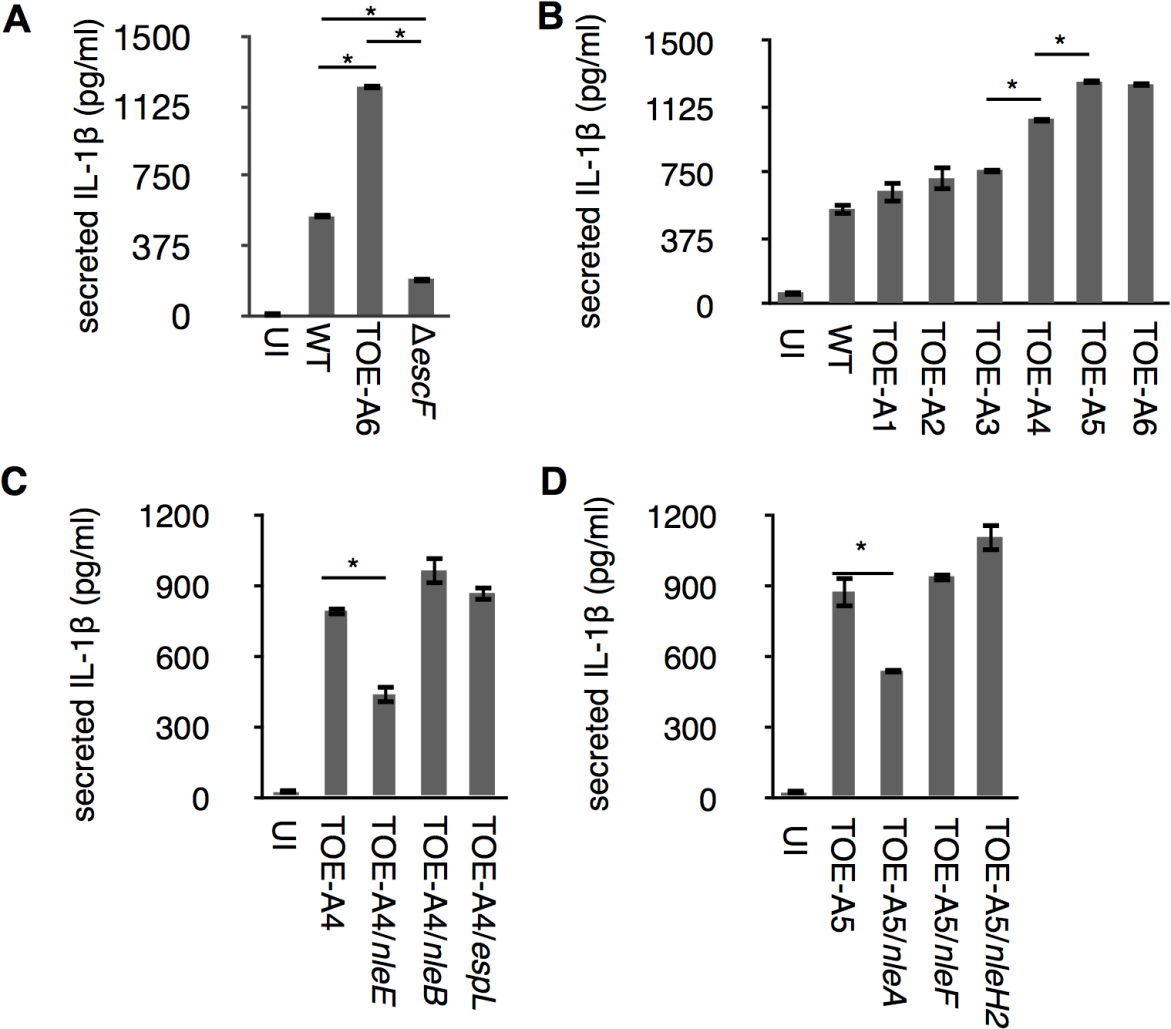
Identification of NleA and NleE as inhibitors of host IL-1β secretion. Differentiated THP-1 cells were infected for 1 hr followed by addition of gentamicin to terminate extracellular bacteria. Cells were further incubated for 6 hrs, and culture supernatant was collected for ELISA analysis. (A) IL-1β secretion from EPEC-infected cells. Cells were uninfected or infected with WT, Δ*escF* (T3SS-deficient strain) and TOE-A6 (non-LEE effector-deleted strain), and the amount of IL-1β in the culture supernatant was measured. (B) TOE-A4 and TOE-A5 isogenic mutants lost their inhibitory property on IL-1β secretion. Cells were infected with WT and TOE-A strains (A1-A6), followed by measurement of the amount of IL-1β secreted in culture supernatants. (C and D) Complementation test for TOE-A4 or TOE-A5 identified NleE and NleA as inhibitors. TOE-A4 possessing *nleE*, or *nleB*, or *espL* were used for infection of THP-1 cells along with the parental strain (C). Alternatively, TOE-A5 possessing *nleA*, or *nleF*, or *nleH2* were used for infection of THP-1 cells along with the parental strain (D). * *p* < 0.05 by Student’s t-test. Experiments were done in triplicate and repeated independently three times for (A) and (B); two times for (C) and (D); and one of the representative experiments with similar results is shown.

The inflammasome plays a major role in the secretion of IL-1β, one of the essential inflammatory cytokines for host defenses against enteropathogens. To determine whether pathogenic *Escherichia coli* could modulate inflammasome activity with effectors, we first induced THP-1 to differentiate into a macrophage-like cells and primed cells with LPS to promote synthesis of pro-IL-1β. LPS-primed dTHP-1 (differentiated THP-1) were uninfected (UI) or infected with wild type EPEC E2348/69 (WT), a Δ*escF* isogenic mutant (T3SS-defective strain), and TOE-A6 (an E2348/69 strain that lacks all non-LEE-effector genes); we then measured the amount of IL-1β using an ELISA assay. Compared with WT, we found that although the Δ*escF* mutant reduced the amount of secreted cytokine, TOE-A6 caused an increase in IL-1β secretion (Fig 1A), despite of comparatively lesser degree of bacterial exposure of TOE-A6 (S1 Fig). The reduction of IL-1β secretion from the Δ*escF* mutant-infected cells is consistent with previous reports in which the T3SS of EPEC/EHEC could elicit an inflammasome response [20]. The significant increase in IL-1β release from the cells infected by TOE-A6 suggested that deleted non-LEE effector(s) might participate in the suppression. To elucidate potential inhibitory effector(s), we performed a screening experiment by infecting dTHP-1 with the EPEC derivatives TOE-A1 to TOE-A6, which are strains with serial deletions of clusters of effector genes [21]. After 1 hr of infection followed by 6 hrs of further incubation, we used an ELISA method to measure the IL-1β secretion from cells infected with TOE-A1 to TOE-A6 strains. We found that there was a significant increase in the TOE-A4 and TOE-A5 strains compared with TOE-A3 and TOE-A4, respectively (Fig 1B). TOE-A4 was derived from TOE-A3 by the deletion of the IE6 region, which contains a cluster of effector genes (*nleE*, *nleB1*, and *espL)*, resulting in a lack of a total of nine effector genes (*nleB2*, *nleH1*, *espJ*, *nleG*, *nleC*, *nleD*, *nleE*, *nleB1* and *espL*). TOE-A5 was generated upon the removal of PP6 from TOE-A4, leading to additional loss in the effector genes *nleH*, *nleA*, and *nleF*. The results of the screening suggested that some of *nleE*, *nleB1*, *espL*, *nleH*, *nleA*, and *nleF* might be involved in the interference of IL-1β secretion. To identify the inhibitory effectors, we performed the rescue test by using plasmids expressing one of these effectors. Plasmids harboring each individual effector gene with a FLAGx3 epitope at the C-terminus were introduced into TOE-A4 or TOE-A5, yielding TOE-A4/pFLAG3*-NleE* (TOE-A4/*nleE*), TOE-A4/ pFLAG3*-nleB1* (TOE-A4/*nleB1*), TOE-A4/ pFLAG3*-espL* (TOE-A4/*espL*), TOE-A5/ pFLAG3*-nleH* (TOE-A5/*nleH*), TOE-A5/ pFLAG3*-nleA* (TOE-A5/*nleA*), and TOE-A5/ pFLAG3*-nleF* (TOE-A5/*nleF*). We then infected dTHP-1 with these established strains and compared the level of IL-1β secretion to their respective parent strains. As shown in Fig 1C and 1D, TOE-A4/*nleE* and TOE-A5/*nleA* exhibited significant suppression of IL-1β release from the host. The expression of other effectors in TOE-A4 or TOE-A5 failed to reduce the secretion levels. These results indicated that at least two non-LEE-effectors, NleA and NleE, are capable of inhibiting host IL-1β secretion.

### NleA suppresses caspase-1 activation

**Fig 2:**
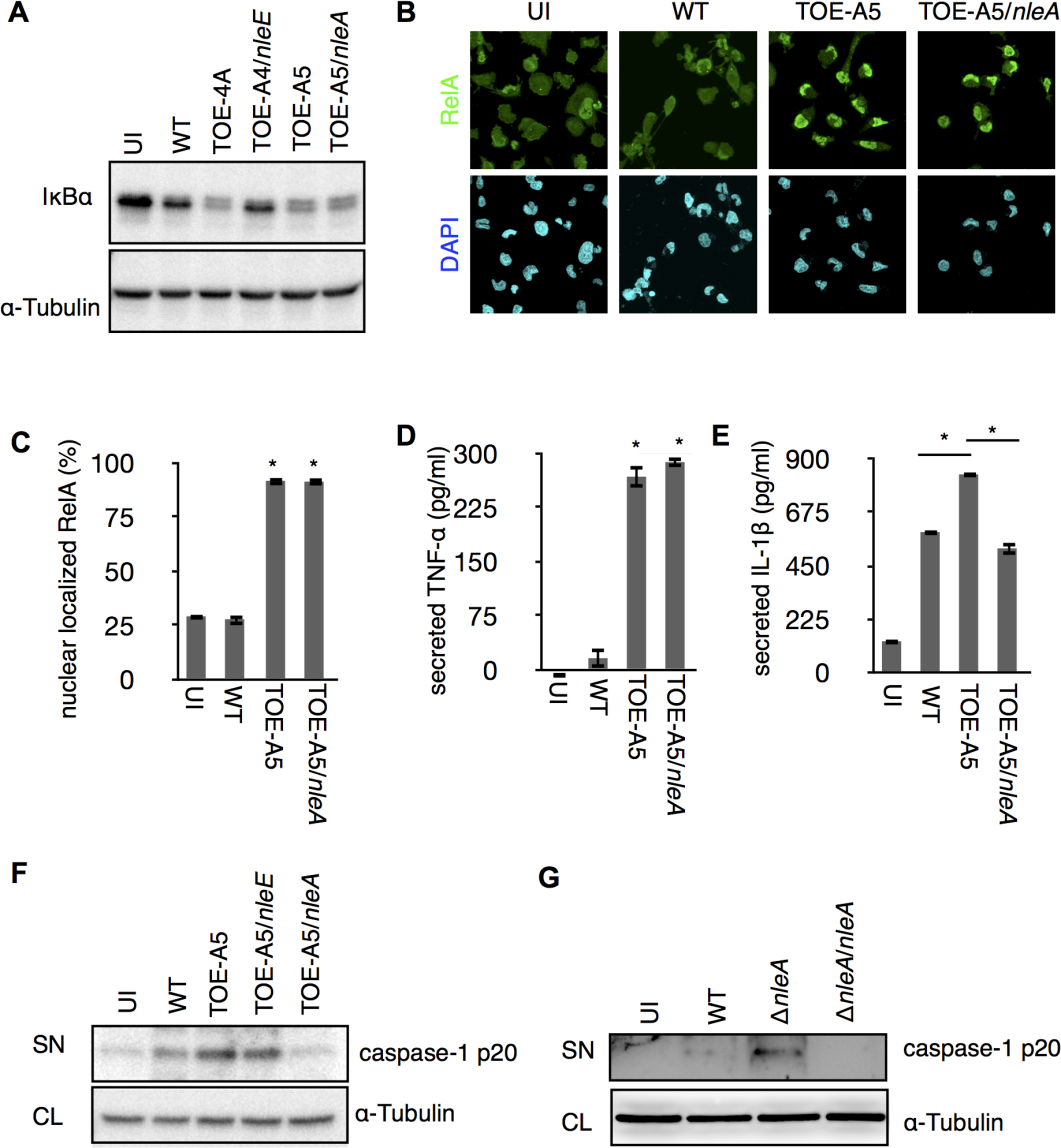
NleA suppresses caspase-1 activation. (A) NleA does not interrupt IκB degradation during bacterial infection. dTHP-1 were uninfected or infected with indicated strains as described in the section of Method with modifications. Cells were centrifuged for 10 min and infected for 1.5 hrs. Then cells were washed once with PBS and directly lysed in 1x SDS sampler buffer. Proteins were separated by SDS-PAGE and probed with anti-IκB and anti-α-tubulin. (B and C) NleA does not inhibit NF-κB nuclear translocation. Differentiated THP-1 cells were uninfected (UI) or infected with wild type (WT), TOE-A5, and TOE-A5/*nleA* strains. After 1 hr of infection and 30 min of gentamicin-treatment, cells were washed, fixed and immunostained with anti-RelA antibody (green) and DAPI (blue). Images from nine random fields were captured using confocal microscopy. Cells with intense nuclear staining of RelA were counted. The percentage of such cells was calculated by dividing the total number of cells in each given field of view. Student’s t-test * *p* < 0.05 (v.s WT). (D and E) NleA inhibits secretion of IL-1β but not TNF-α. Differentiated THP-1 cells were uninfected (UI) or were infected by WT, TOE-A5, and TOE-A5/*nleA* for 1 hr, and the culture supernatants at 6 hr post-infection were analyzed by ELISA. Student’s t-test * *p* < 0.05 (D: vs WT). (F and G) NleA inhibits caspase-1 activation. After 1 hr of infection followed by (F) 6 hrs or (G) 3 hrs of incubation, culture supernatant from cells uninfected (UI) or infected with indicated strains were centrifuged and the proteins in the supernatant were TCA-precipitated. Immunoblottings were performed to assess the amount of active caspase-1 (caspase-1 p20) by anti-caspase-1 antibody. SN, supernatant; CL, cell lysate.

The *IL1B* gene is a regulatory target of NF-κB transcription factors (NF-κBs), and the synthesized cytokine requires enzymatic processing by active caspase-1 for secretion [22]. We found a significant reduction of cellular pro-IL-1β in the presence of NleE but not NleA in infected cells (S2 Fig). Because NleE has been known to be a potent inhibitor of the NF-κB pathway [23–26], it is likely that NleE reduces IL-1β production by inhibiting the activation of NF-κB. Indeed, we found infection of cells with the pathogen expressing NleE hampers the IκB degradation, which is necessary for the activation of NF-κB, but such inhibition was not seen in cells infected with NleA-expressing strain (Fig 2A). We focused on NleA because this effector has not yet been reported to be involved in the regulation of host inflammation. In a steady state, NF-κBs are inhibited by IκB (Inhibitor of κB) in the cytoplasm; however, the NF-κBs are translocated into the nucleus after IκB is degraded upon immune challenge [27]. We further explored the effect of NleA on nuclear translocation of RelA, a subunit of NF-κBs in the infected cells. By immunostaining the cells that were infected with WT, TOE-A5, and TOE-A5/*nleA*, we found the WT-infected cells had relatively weak nuclear RelA staining, which is in accordance with previous observations [21]. The majority of the cells infected with TOE-A5 or TOE-A5/*nleA* exhibited strong nuclear RelA staining (Fig 2B). By enumerating the percentage of cells showing strong nuclear RelA signals, we concluded that NleA does not affect RelA nuclear translocation (Fig 2C). Finally, we examined the effect of NleA on the ability of NF-κBs to produce other inflammatory cytokines. We infected dTHP-1 with WT, TOE-A5, and TOE-A5/*nleA* and measured the concentrations of TNF-α, another NF-κB-dependent cytokine, and IL-1β from the identical cell culture medium. We found that TNF-α secretion was unaffected in the cells infected with TOE-A5/*nleA* compared with the cells infected with TOE-A5 (Fig 2D). A significant impediment in IL-1β secretion occurred in the cells infected with TOE-A5/*nleA* and not in the cells infected with TOE-A5 (Fig 2E).

Unlike TNF-α, IL-1β secretion is independent of the conventional ER-Golgi secretion pathway and requires additional enzymatic cleavage by active caspase-1 [28]. Therefore, we speculated that NleA might negatively influence IL-1β-specific processing or secretion pathways, such as the activation of caspase-1. To examine this possibility, we infected dTHP-1 with WT, TOE-A5, TOE-A5/*nleE*, and TOE-A5/*nleA* and analyzed the p20 subunit of active caspase-1 in the cell culture medium by immunoblotting. As shown in Fig 2E, the p20 subunit was noticeably decreased in the cells infected with TOE-A5/*nleA* compared with the cells infected with TOE-A5 (Fig 2F). To further confirm that NleA alone is sufficient for the reduced caspase-1 activation, we infected dTHP-1 with WT, Δ*nleA*, and the *nleA* complemented strain Δ*nleA/nleA*. We found that the EPEC lacking *nleA* gene resulted in increase of caspase-1 p20 in the culture medium compared to WT, and that such increase was diminished by introducing the *nleA* gene (Fig 2G). Taken together, these results suggest that NleA might directly or indirectly downregulate caspase-1 activation, and less active caspase-1 contributes to less processing of IL-1β for secretion. Unlike NleA, NleE did not show an effect on the production of active caspase-1, indicating that NleA reduces IL-1β secretion through a mechanism completely different from that of NleE. These results showed that NleA did not interfere with the NF-κB pathway; however, NleA reduces the amount of active caspase-1 required for IL-1β secretion.

### NleA suppresses inflammasome formation

**Fig 3:**
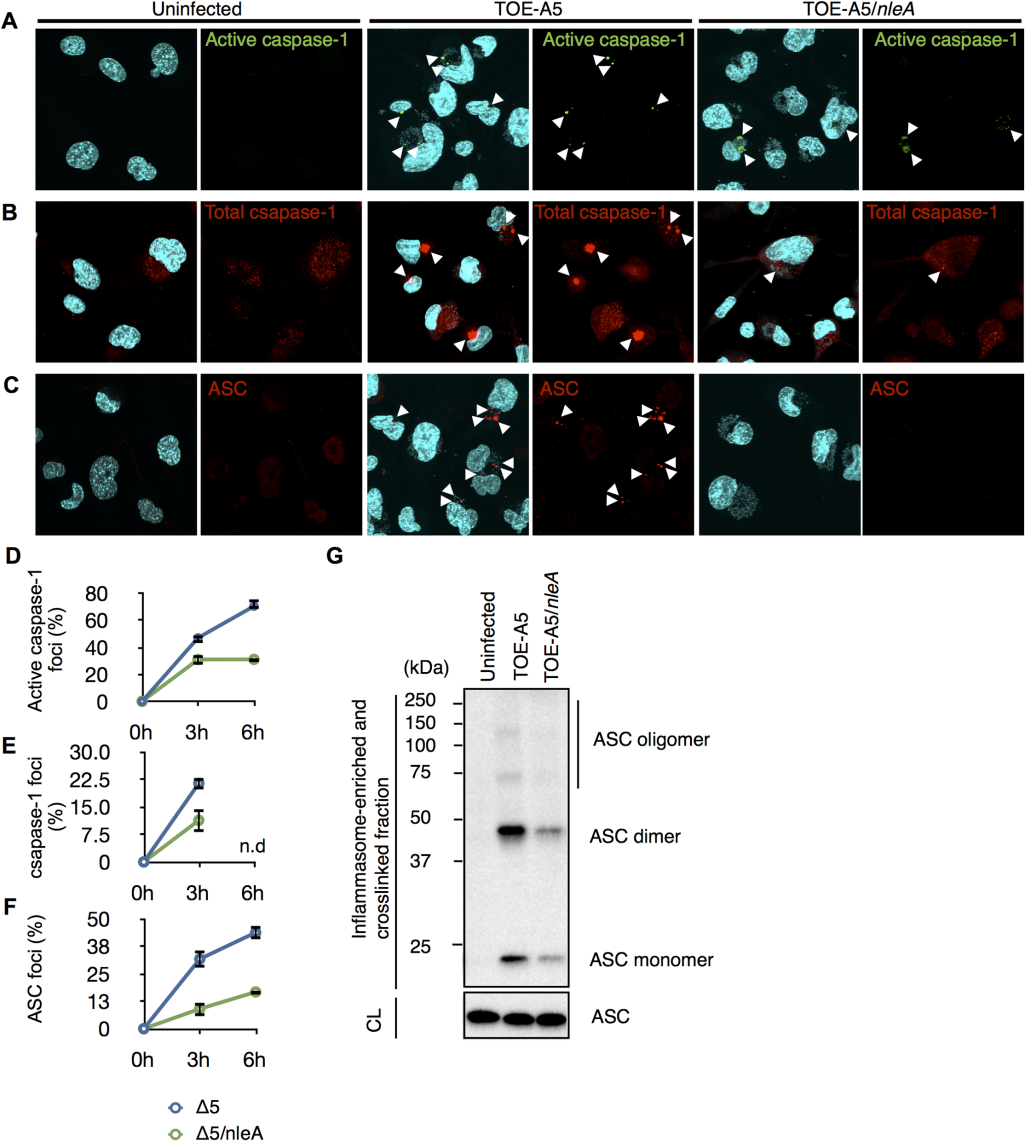
NleA suppresses formation of the inflammasome. Differentiated THP-1 cells were uninfected (UI) or infected with TOE-A5 and TOE-A5/*nleA* for 1 hr and further incubated for 3 and 6 hrs. Cells at the indicated times were immunostained, followed by visualization using a confocal microscope. The foci are indicated by arrowheads. (A) Foci formed with active caspase-1 in infected cells. The cells were stained with FAM-YVAD-FMK FLICA for active caspase-1 (Green) and DAPI (Blue). (B) Foci formed with total caspase-1 in infected cells. The cells were stained with anti-caspase-1 (Red) and DAPI (Blue). (C) Foci formed with ASC in infected cells. The cells were immunostained with anti-ASC (Red) and DAPI (Blue). (D, E and F) Quantification of respective foci formation. From nine random fields, the percentages of active caspase-1-, total caspase-1-, or ASC- foci were calculated as (number of identified speck-like structures)/(number of cells in a given field of view)x100%. Student’s t-test, * *p* < 0.05. n.d, not determined. (G) NleA reduces ASC oligomer formation. After 1 hr of infection and 3 hrs of further incubation, the cell lysates from uninfected (UI) or cells infected with TOE-A5 or TOE-A5/*nleA* were enriched for the inflammasome-containing fraction. The fraction was further treated with a DSS protein-protein cross-linker for 30 min at room temperature, and the reaction was terminated by the direct addition of 2x SDS sample buffer. The samples were separated by SDS-PAGE, and an anti-ASC antibody was used to detect ASC species. CL, cytosolic lysate.

The observation of reduced active caspase-1 by NleA prompted us to further investigate the mechanism by which this effector affects the processes leading to the generation of active caspase-1, predominantly the inflammasome pathway. Active caspase-1 is generated from the autoprocessing of its precursor protein (pro-caspase-1) following recruitment into the inflammasome complex [29,30]. Because the inflammasome consists of the NLR protein, ASC, and caspase-1, it could be visualized as a speckled structure or foci after the immunofluorescent staining of its constituent proteins [31]. We first examined the number of formed active caspase-1 foci in the uninfected (UI), TOE-A5-infected, and TOE-A5/*nleA*-infected dTHP-1 cells at 3 and 6 hrs post-infection. Labeling the infected cells with FAM-YVAD-FMK, a fluorescent irreversible inhibitor probe of active caspase-1, revealed minimal formation of foci in the uninfected dTHP-1 cells and a comparatively higher number of foci in cells infected by TOE-A5 (Fig 3A). This result is consistent with the finding that infection with TOE-A5 increased the p20 subunit production (Fig 2E). However, when comparing the TOE-A5/*nleA*- infected dTHP-1 cells to the cells infected with TOE-A5, we observed significantly fewer foci in the presence of NleA (Fig 3A and 3D). This observation is consistent with the result of reduced active caspase-1 production by TOE-A5/*nleA*-infected cells. Next, we examined the inflammasome that contains any form of caspase-1 (Pro- and active forms) by immunofluorescent staining with an anti-caspase-1 antibody. As shown in Fig 3B and 3E, the number of foci containing total caspase-1 foci was consistently lower in the dTHP-1 cells infected with TOE-A5/*nleA* than in the cells infected with TOE-A5 (Fig 3B and 3D), indicating that NleA blocks the recruitment of pro-caspase-1 into the inflammasome. Because ASC functions in the formation of the inflammasome by bridging NLRP and caspase-1, we next examined the ASC foci in the infected dTHP-1 cells. As shown in Fig 3C and 3F, fewer ASC foci were observed in the TOE-A5/*nleA*-infected cells than in the TOE-A5-infected cells. Fernandes-Alnemri *et al*. previously showed that the stimulation and activation of the inflammasome leads to the formation of a large ASC oligomer complex, and this complex could be fractionated by centrifugation [32]. To compare the formation of such complexes, we isolated the complexes by the centrifugation of the lysates of uninfected, TOE-A5-, and TOE-A5/*nleA*-infected cells following protein-protein cross-linkage. As shown in Fig 3G, we observed a decrease in the total amount of ASC and ASC-dimers and oligomers in complexes in TOE-A5/*nleA*-infected cells compared with the TOE-A5-infected cells (Fig 3G). These results indicated that NleA hinders the formation of the ASC-containing inflammasomes and their oligomerization. We showed that NleA causes a reduction in active caspase-1 by prohibiting the formation of the inflammasome.

### NleA interferes with the activation and formation of the NLRP3 inflammasome

**Fig 4:**
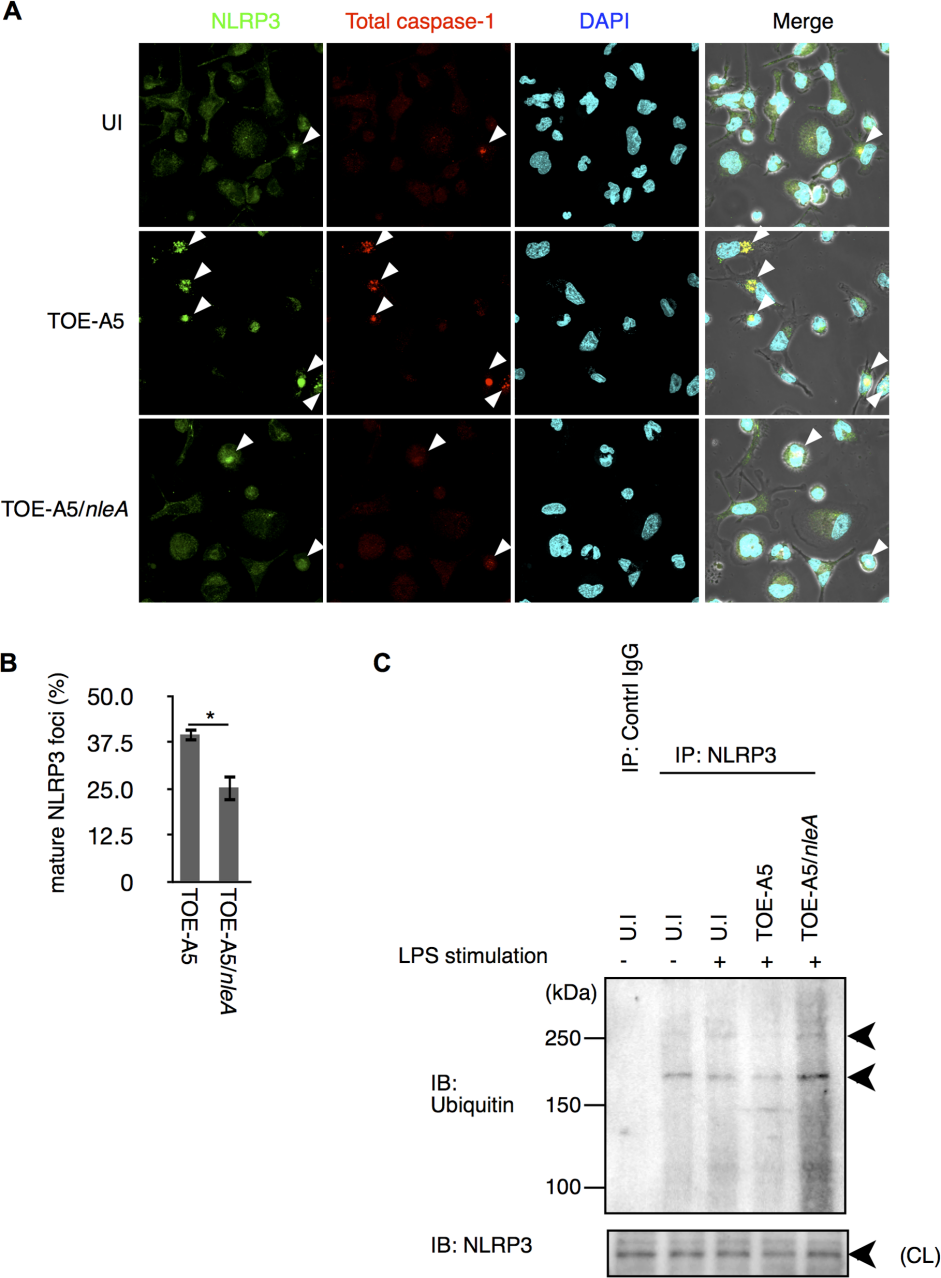
NleA reduces formation of mature NLRP3 inflammasomes. (A) Mature NLRP3 foci in infected THP-1 cells. Differentiated THP-1 cells were uninfected (UI) or infected with TOE-A5 or TOE-A5/*nleA*. After 1 hr of infection and 3 hrs of further incubation, the cells were processed for immunostaining with an anti-NLRP3 antibody (Green), total caspase-1 (Red) and DAPI (Blue). A mature NLRP3 focus was identified as strong co-localization of the signals between NLRP3 and total caspase-1 (arrowheads). (B) Quantification of mature NLRP3 foci. Using a confocal microscope, nine random fields were investigated, and the number of mature NLRP3 foci was counted. The percentage of foci was expressed as (number of identified speck-like structures)/(number of cells in a given field of view) x100%. Student’s t-test, * *p* < 0.05. (C) NleA interferes with the de-ubiquitin modification of NLRP3. Differentiated THP-1 cells were primed with LPS (1 μg/ml) for 2 hrs prior to infection. Cells were then uninfected (UI) or infected with TOE-A5 or TOE-A5/*nleA* for 1 hr and then incubated for 3 hrs. The cell lysates were subjected to immunoprecipitation by an anti-NLRP3 antibody, and the precipitated products were analyzed by immunoblotting with an anti-ubiquitin antibody. NLPR3 in cell lysate (CL) used for immunoprecipitation was also detected.

The NLRP3 inflammasome is known to be triggered by infection with A/E pathogens and shows a non-redundant role in caspase-1 activation when mouse macrophages (mBMDM) are challenged with *Citrobacter rodentium*, a mouse A/E pathogen possessing the *nleA* homologous gene [19]. Therefore, we hypothesized whether NleA might limit NLRP3 inflammasome formation. After infecting dTHP-1 cells with TOE-A5 or TOE-A5/*nleA* for 1 hr followed by 3 hrs of incubation, the cells were co-immunostained with antibodies against NLRP3 and total caspase-1 to detect the mature NLRP3 inflammasome. We observed fewer NLRP3 inflammasomes in the cells infected with TOE-A5/*nleA* than in those infected with TOE-A5 (Fig 4A and 4B). The majority of the caspase-1 foci overlapped with the NLRP3 foci. This result suggested that EPEC infection induces the formation of the NLRP3 inflammasome in human macrophage-like cells; however, NleA negatively regulates the formation of the NLRP3 inflammasome.

Ubiquitin modification controls NLRP3 protein activity [33,34]. It has been shown that primary or secondary signal alone can each trigger certain degree of de-ubiquitination in NLRP3 without caspase-1 activation; and, it is the combination of two signals induce the greatest extend of de-ubiquitination leading to the assembly of the NLRP3 inflammasome and caspae-1 processing, reflecting the nature of strict regulations on this pathway [33]. We found that cells stimulated with LPS for 15 min or 2 hrs had reduction in ubiquitinated NLRP3, but still retained a significant amount (S3 Fig). Furthermore, these LPS treatments alone produced below the detection level of caspase-1 processing. Because TOE-A5/*nleA* infection inhibited the assembly of the NLRP3 inflammasome, we examined the degree of de-ubiquitination of NLRP3. The cells were treated with LPS for 2 hrs and then infected with bacteria for 1 hr, followed by 3 hrs of incubation. The endogenous NLRP3 were isolated by immunoprecipitation using an anti-NLRP3 antibody, and the ubiquitinated NLRP3 were detected with an anti-ubiquitin antibody. As shown in Fig 4C, compared to uninfected cells, while the infection of TOE-A5 caused reduction in ubiquitinated NLRP3, the amount of the ubiquitinated NLRP3 in the TOE-A5/*nleA*-infected cells did not decrease but increased, indicating that NleA directly or indirectly influence this step of inflammasome activation. We also tested whether NleA can hinder the NLRP3 inflammasome activation in infected cells treated with nigericin, an agonist of NLRP3 inflammasome. We found that the elicitation of caspase-1 activation to be less when NleA was available (S4 Fig). Taken together, these results showed that NleA specifically targets NLRP3 inflammasome activation eliciated by infection and that the effector prevents the assembly of the NLRP3 inflammasome, possibly through interfering the change of ubiquitination in NLRP3 during infection.

### NleA directly targets NLRP3

**Fig 5:**
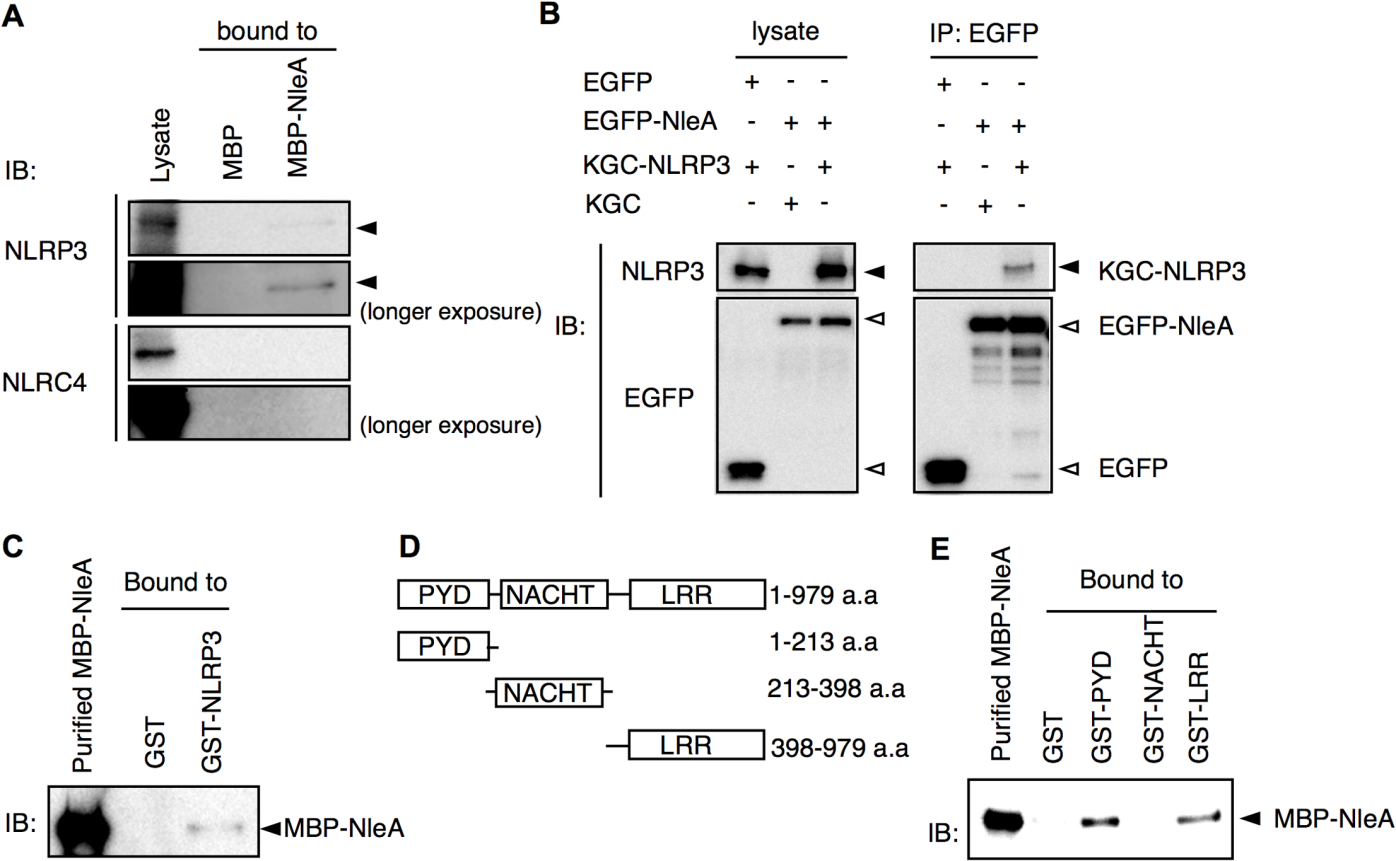
NLRP3 directly interacts with NleA. (A) NleA associates with endogenous NLRP3. Columns packed with resins of bacterial expressed MBP (8 μg) or MBP-NleA (8 μg) were used to pulldown endogenous proteins of the THP-1 lysates prepared from approximately 1x10^8^ cells. The bound proteins were eluted with a high salt elution buffer and analyzed with the indicated antibodies. (B) NleA interacts with NLRP3 in cells. HeLa cells were seeded in 6-well plates and transfected with plasmids expressing the indicated fusion proteins for 24 hrs. The cell lysates were immunoprecipitated (IP) with anti-GFP-coupled magnetic beads. The pulldown products were analyzed by immunoblotting (IB) using anti-NLRP3 and anti-GFP antibodies. (C) NleA directly binds NLRP3. Purified GST or GST-NLRP3-bound resins (2.5 μg each) were packed in columns to pull down the purified MBP-NleA (250 μl of 5 μg/ml). The bound proteins were eluted with a high salt elution buffer and analyzed by IB using an anti-MBP antibody. (D) Domains of NLRP3. The amino acid sequences containing one of three domains were fused with GST for purification. (E) NleA binds to the PYD and LRR domains of NLRP3. GST or GST-PYD, GST-NACHT, and GST-LRR fusion proteins were purified and immobilized on resins at approximately 2.5 μg each. An equal amount of purified MBP-NleA (500 μl of 5 μg/ml) was applied for direct binding. The columns were extensively washed with column buffer. The eluted products were probed with an anti-MBP antibody.

To identify potential targets of NleA involved in the inflammasome pathways, a cytosolic extract of THP-1 was applied through columns packed with purified MBP or MBP-NleA-bound resins. Potential inflammasome-related proteins were analyzed with specific antibodies by immunoblotting. We found, upon probing with anti-NLRP3, the immunoblot revealed that NLRP3 specifically interacts with MBP-NleA. We failed to detect NLRC4, which is another activator of caspase-1, in the NleA-bound fraction. This result indicated that NleA interacts with NLRP3 and not with NLRC4 (Fig 5A). To further validate the association between NleA and NLRP3, we co-transfected HeLa cells with plasmids expressing EGFP-NleA and KGC-NLRP3 and performed a co-immunoprecipitation assay. As shown in Fig 5B, EGFP-NleA, but not EGFP, was able to interact with KGC-NLRP3, confirming the association between NleA and NLRP3. To further determine whether this interaction is direct, we examined the binding of a purified MBP-NleA fusion protein with a purified GST-NLRP3 fusion protein. To perform the assay, we applied MBP-NleA to columns packed with GST or GST-NLRP3-bound resins. We found that GST-NLRP3, but not GST, could interact with MBP-NleA, indicating a direct interaction between the effector and the host factor (Fig 5C). NLRP3 consists of the following three domains: the PYD, NACHT, and LRR domains. Additionally, to determine the domain of NLRP3 that NleA binds, we constructed bacterial plasmids that express GST-PYD, GST-NACHT, and GST-LRR (Fig 5D). After purification and immobilization on resins in the columns, purified MBP-NleA was applied to each column. After extensive washing and the elution of the bound proteins, we analyzed the presence of MBP-NleA in the eluates of these columns by immunoblotting; we found that MBP-NleA interacts with the GST-PYD and GST-LRR constructs (Fig 5E). These results indicate that NleA could directly target NLRP3 through interactions with the PYD and LRR domains.

### NleA prevents de-ubiquitination of NLRP3

**Fig 6:**
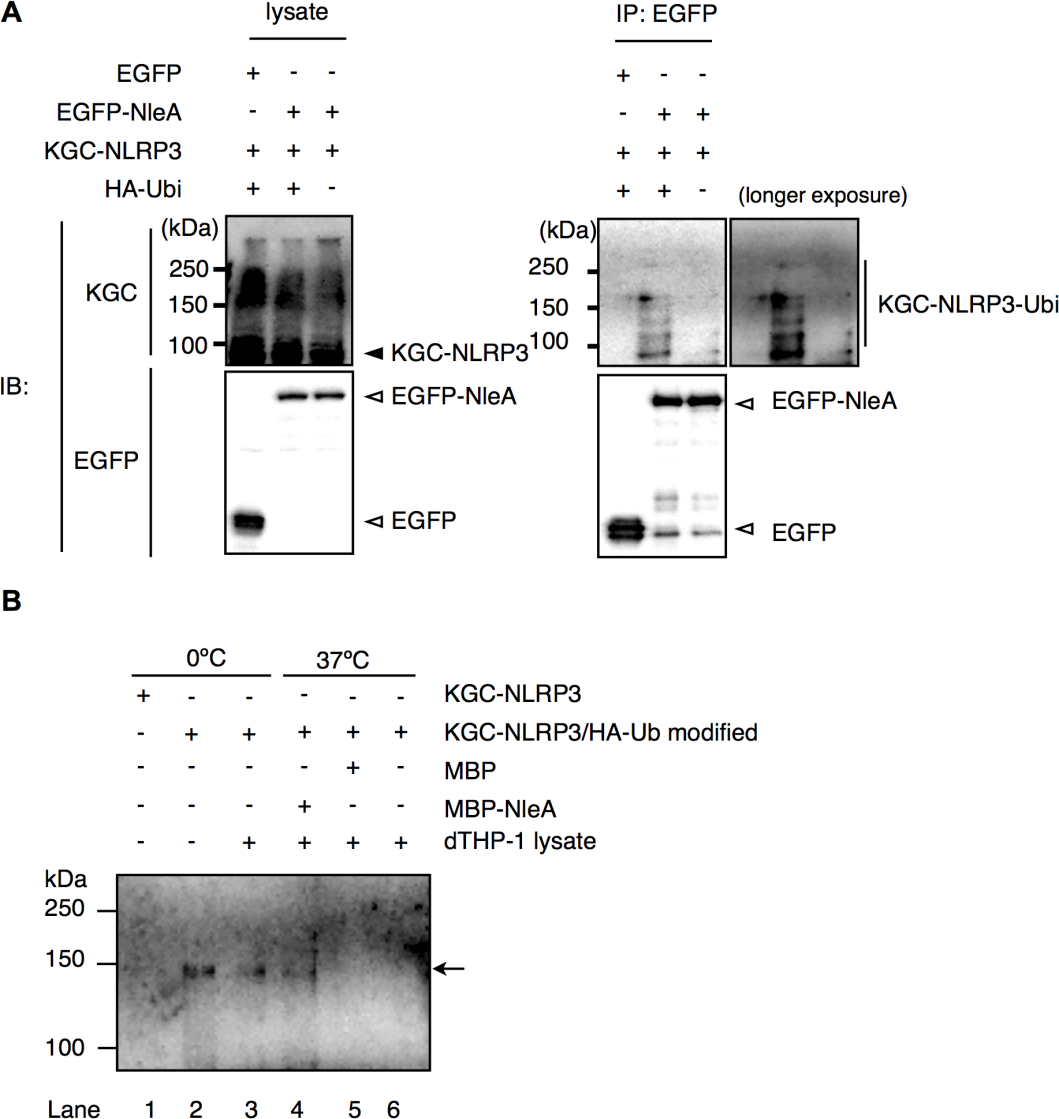
NleA binds to ubiquitinated NLRP3 and represses de-ubiquitination. (A) NleA binds to ubiquitinated NLRP3. Lysates of HeLa cells expressing combinations of proteins as indicated were subjected to immunoprecipitation by an anti-GFP antibody, and the NLRP3 fusion proteins and GFP fusion proteins were detected by immunoblotting using anti-KGC and anti-GFP antibodies. (B) In vitro de-ubiquitination assay of NLRP3. The total KGC-NLRP3 (including HA-ubiquitin modified KGC-NLRP3) was purified and immobilized on magnetic beads using an anti-KGC antibody. Non-treated or MBP- or MBP-NleA-treated immobilized KGC-NLRP3 was resuspended in the assay buffer and LPS-primed dTHP-1 lysate was added at 1/100 of reaction volume. The reaction was performed at 0°C or 37°C for 1 hr and was terminated by first washing the beads with the assay buffer, followed by the addition of 1x SDS sampler buffer. The amount of HA-ubiquitinated NLRP3 was analyzed by immunoblot using an anti-HA antibody. Please refer to the Methods section for more detailed descriptions.

In unstimulated cells, NLRP3 is ubiquitinated; however, NLRP3 undergoes de-ubiquitination upon LPS stimulation [33]. Because infections with NleA-producing bacteria reduced the de-ubiquitination of NLRP3 compared with that of the NleA-negative bacteria, it is likely that NleA binds to ubiquitinated NLRP3 and inhibits de-ubiquitination. In prior co-immunoprecipitation assays between EGFP-NleA and KGC-NLRP3 (Fig 5B), we did not observe any higher-molecular-weight KGC-NLRP3. This result might be because of an insufficient amount of ubiquitinated KGC-NLRP3 in the transfected cells. To improve these results, we scaled up the transfection and expressed KGC-NLRP3 with or without HA-ubiquitin and EGFP or EGFP-NleA in HeLa cells. Cell lysates were immunoprecipitated with an anti-GFP antibody; the pulldown products were probed with an antibody against a KGC-epitope to detect the total KGC-NLRP3. As shown in Fig 6A, higher-molecular-weight KGC-NLRP3 was co-precipitated with EGFP-NleA from the lysate of HA-ubiquitin expressing cells. In addition, KGC-NLRP3-Ubi was detected in the pulldown products from cells without HA-ubiquitin, although the amount was much smaller. Given that NleA reduces the de-ubiquitination of NLRP3 during infection and that NleA binds to ubiquitinated NLRP3 in a co-immunoprecipitation assay, we speculated that NleA could inhibit the de-ubiquitination of NLRP3 upon stimulation. To verify the inhibitory effect of NleA on NLRP3 de-ubiquitination, we conducted an in vitro de-ubiquitination assay. Total KGC-NLRP3 proteins were purified from the plasmid-transfected HeLa cells either expressing KGC-NLRP3 alone or co-expressing KGC-NLRP3 and HA-ubiquitin, and were immobilized on magnetic beads with an anti-KGC antibody. The beads were pre-mixed with assay buffer or MBP or MBP-NleA followed by addition of the cell lysates extracted from the LPS-primed dTHP-1 cells. The reaction was performed at 0°C or 37°C for 1hr before being terminated with SDS sample buffer. Samples were analyzed by anti-HA antibody to detect changes in HA-ubiquitinated KGC-NLRP3. Incubation with dTHP-1 lysate greatly reduced the HA-ubiquitinated KGC-NLRP3 in samples pre-incubated with the buffer or MBP, while, the sample pre-incubated with MBP-NleA had partially lost HA-ubiquitinated KGC-NLRP3 but the degree was not as extensive as those of buffer- or MBP-treated sample (Fig 6B, compare Lane 4 to lanes 5 and 6). Taken together, these results strongly suggest that NleA can bind to both non- and ubiquitinated NLRP3 and that it interferes the alteration of the amount of ubiquitinated NLRP3 in infected cells.

## Discussion

The sensor molecule NLRP3 and its family members are central to the inflammasome in response to a wide range of stimuli, whereby are responsible for the activation of caspase-1 and the secretion of inflammatory IL-1β and related cytokines for amplifying inflammation. Prior studies have demonstrated that infections with EPEC, EHEC and *Citrobacter rodentium* could elicit inflammasomes in macrophages and epithelial cells [17,19], but our study is the first to show that A/E pathogens could actively suppress IL-1β production and that a T3SS-dependent effector, NleA, is used to inhibit host inflammasome formation in macrophage-like cells.

NF-κB and the inflammasome regulate transcription and secretion of IL-1β, respectively [22]. Interference in each or both pathways will result in an overall reduction in the output of IL-1β. We initially suspected that the TOE-A4 strain would lose suppression of IL-1β production because most of the reported NF-κB suppressors, including NleC, NleB, NleE, and EspL are absent. Significant de-repression was seen in TOE-A4, which is similar to that observed in our previous study [21]. The subsequent screening of effectors restoring TOE-A4 (TOE-A4/*nleE*, TOE-A4/*nleB*, and TOE-A4/*espL*) only indicated that NleE inhibited IL-1β.This finding may be because cells were only being challenged by added pathogens during infection, instead of the addition of other stimuli such as TNF-α, whose signal pathway has been shown to be inhibited specifically by NleB [24]. Because bacterial effectors are versatile and frequently possess multi-functions, we investigated whether NleE affects the inflammasome pathway by examining the amount of active caspase-1 in infected cells. The activation of caspase-1 appeared to be unhindered by the presence of NleE, implicating that the suppression of NF-κB is likely the determinant event in the NleE-mediated reduction of IL-1β.

NLRP3 and NLRC4 are the most frequently studied NLRs, and they each respond to different sets of agonists. Upon activation, NLRP3 depends on ASC to assemble a mature inflammasome, whereas NLRC4 could directly bind and activate pro-caspase-1 [35]. In bacterial infections, there appears to be a cell-context dependent requirement of NLRC4 in protection from A/E pathogen infection, whereas Nordlander *et al*. showed that NLRC4 is dispensable for eliciting caspase-1 activation in macrophages, Liu *et al*. showed that NLRC4 is pivotal for caspase-1 activity in non-hematopoietic cells [19,36]. Infection of the mouse A/E pathogen *C*. *rodentium* has been shown to activate the NLRP3 inflammasome, which is critical for the production of IL-1β and IL-18 and protection in mouse macrophages [19]. In our initial study, we found that the deletion of *escF*, a major component of the T3SS injectosome, renders the mutant strain inert to stimulate IL-1β secretion from THP-1 cells, which implicates the contribution of NLRC4 to host cell defense [20]. However, in our pulldown assay using MBP-NleA and THP-1 lysates, we found that the effector associates with NLRP3 and that it does not associate with NLRC4. Because infection of THP-1 cells with EPEC induced the formation of the NLRP3 inflammasome and NleA reduces the formation of the caspase-1 containing inflammasome, activation of the NLRP3 inflammasome might play a critical role in caspase-1 activation in THP-1 cells. Infection of A/E pathogens, such as EPEC and EHEC, might activate NLRC4 and NLRP3 inflammasomes. However, because NLRC4 requires accessory factors to recognize agonists before activation, NleA might target these co-factors, such as NAIP [37]. Prevention of inflammasome formation by NleA contributes to the reduction of the secretion of IL-1β and IL-18 and the inflammatory response of the host.

**Fig 7:**
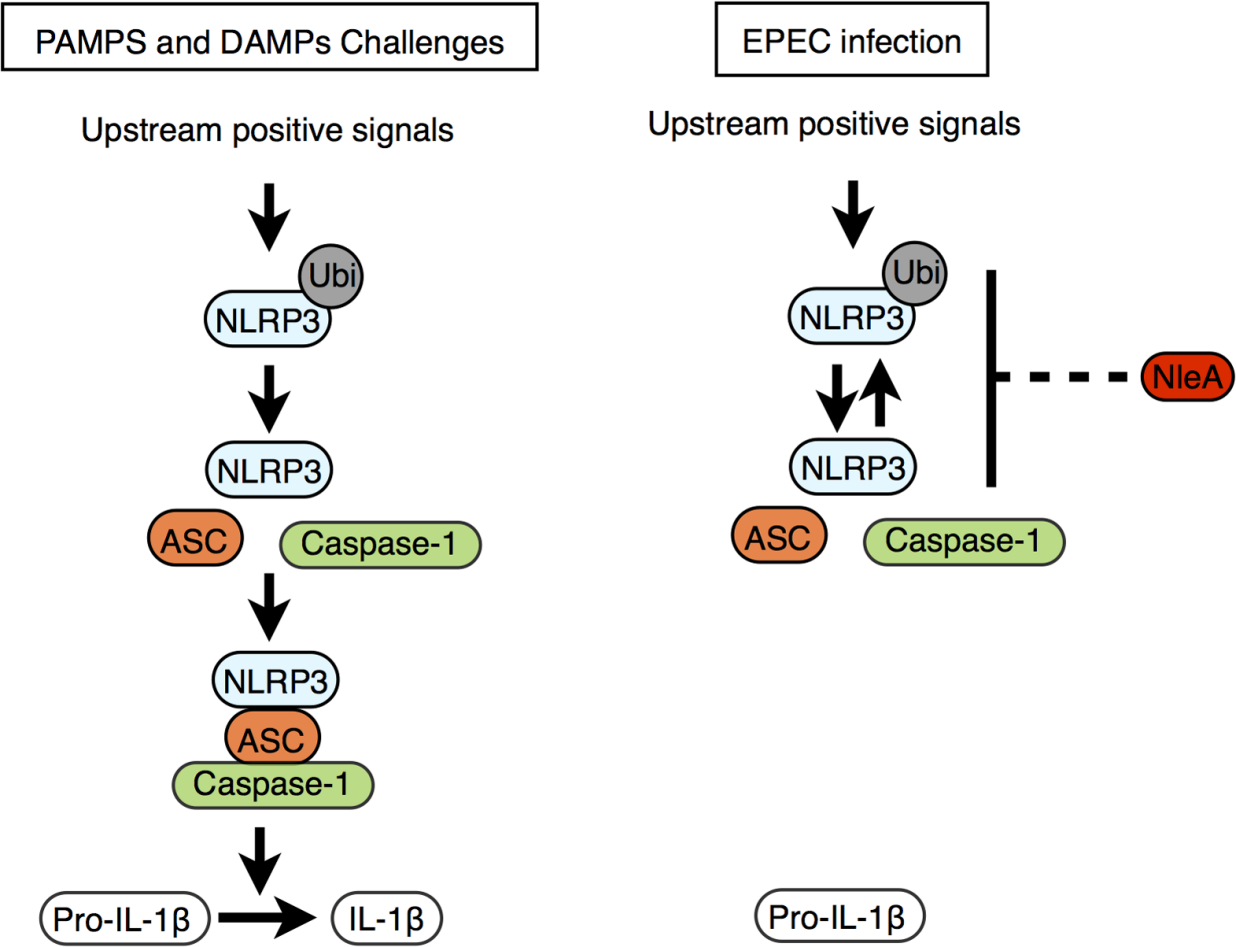
Proposed function of NleA as an inhibitor of NLRP3. Stimuli including PAMPs and DAMPs trigger the onset of various upstream signaling pathways, and these pathways converge to activate the NLRP3 inflammasome (left panel). The upstream positive signals promote the de-ubiquitination of NLRP3 and allow NLRP3 to begin recruitment of ASC and caspase-1. The formed mature NLRP3 inflammasome then becomes competent for enzymatically processing pro-IL-1β into IL-1β by active caspase-1. EPEC and other A/E pathogens use NleA to subvert the normal pathway leading to the assembly of the mature NLRP3 inflammasome. This blockade of the pathway occurs at stage of NLRP3 activation where NleA directly associated ubuiqtinated and non-ubiqutiinated NLRP3, which would prohibit the subsequent inflammasome assembly, resulting in dampening of the processing of pro-IL-1β for secretion.

Recently, Juliana *et al*. showed that the de-ubiquitination of NLRP3 is critical for inflammasome activation following PAMP/DAMP stimulation [33]. This result coincides with our finding that de-ubiquitination in NLRP3 and inflammasome formation were both reduced in cells infected with NleA-expressing EPEC compared with cells infected with non-expressing strains. More ubiquitinated NLRP3 is present when NleA is available. This result suggests that NleA alters the process of ubiquitin modification of NLRP3. Although the precise lysine residues and the degree of ubiquitination in endogenous NLRP3 that might account for the inactivation remain unclear, Py *et al*. demonstrated that a mix of K48- and K63-polyubiquitin chains are present and that modifications occur at regions of the NATCH and LRR but not PYD domains [34]. We found that NleA is able to directly bind to the LRR and PYD domains of NLRP3. Binding to these domains might inhibit access of the de-ubiquitinating enzyme to polyubiquitin at the LRR domain. In addition, because the PYD domain of NLRP3 is responsible for mediating interactions with ASC, it is plausible that NleA might inhibit such an association and prevent further assembly of the NLRP3 inflammasome (Fig 7).

Bacterial subversion of the inflammasome is common among other enteric pathogens. Diverse strategies by effector proteins have been described; for example, YopK of *Yersinia spp*. associates with components of the T3SS to prevent recognition by host sensing molecules [38], and YopM of *Yersinia spp*. and OspC3 of *Shigella spp*. directly bind to inhibit Caspases-1 and -4 activity, respectively [39,40]. In comparison, the inhibitory mechanism of NleA, which directly targets and suppresses the ubiquitination modification of NLRP3, presents a novel strategy among bacterial pathogens. In viral pathogens, however, targeting NLRs has been previously reported; for example, Orf63 of Kaposi’s sarcoma-associated herpes virus and V protein of Measles virus have been reported to target NLRP [41,42]. In particular, Orf63 is a viral homolog of several NLRs proteins and could interact with NLRP3 and with NLRP1, resulting in the inhibition of NLRs oligomerization [41]. However, whether Orf63 could additionally bind to the ubiquitinated form of NLRP and influence changes in the modification is unknown. We found no amino acid sequence homology between NleA and members of NLR proteins or between NleA and Orf63; this result suggests that NleA is a novel type of virulence factor targeting NLRP3. Although they are different in their inhibitory mechanisms, these viral and bacterial proteins have evolved independently to target a common host factor and highlight the critical roles of NLRP3 in host defenses against bacterial and viral pathogens.

NleA has been shown to localize to Golgi [43] and its C-terminal end of 40 amino acids interacts with several members of Sec24 paralogues [43,44]. The association affects COPII formation, ER-Golgi transportation and the maintenance of tight junctions [43–45]. In current study using THP-1 cells, we found that bacteria-delivered NleA-FLAG proteins localized diffusedly throughout cytoplasm with a fraction overlapping with Golgi apparatus (S5 Fig). Furthermore, compared to the full length NleA, we have also shown that NleA lacking C-terminal end of 33 or 62 amino acids is not as effective as the full length in inhibition of caspase-1 activation nor IL-1β secretion (S6 Fig). These findings suggest that the C-terminal end of NleA is involved in the interference of host Caspase-1 activity. At the present time, the precise molecular mechanism responsible for observed reduction in de-ubiquitinated NLRP3 by NleA still remain to be determined. It is plausible that the association of NleA to ubiquitinated NLRP3 hinders the access of host de-ubiquitinase or that the binding of NleA promotes the ubiquitination of NLRP3 by the effector itself or a third unknown factor, or that the association enforces a closed conformation of NLRP3, which remains inactive (Fig 7).

NleA has been shown to be required for infections with *Citrobacter rodentium* in the mouse [46]. A lack of NleA in *Citrobacter rodentium* resulted in attenuated symptoms of milder hyperplasia in the large intestine and in reduced intestinal inflammation. Reduced severity of disease in NleA-deficient strains could be caused by the reduced efficiency of colonization in the colon [46]. Because the absence of NleA resulted in higher IL-1β secretion by macrophages, this might implicate a more efficient inflammatory response from host cells at early stages of infection to increase the clearance of bacteria. Recently Song-Zhao *et al*. and Wlodarksa *et al*. each reported the critical roles of NLRP3 and NLRP6 in intestinal epithelial and goblet cells, respectively, against the infection of enteropathogens [47,48]. Defects in these NLRs results in increase in epithelial colonization of *C*. *rodentium* in infected mouse. Therefore, NleA may, in addition to reported function of disrupting tight junctions, also help increasing the colonization by targeting NLRP3 and potentially NLRP6 in nonhematopoietic cells.

In conclusion, we showed that NleA negatively modulates host NLRP3 inflammasome activity by interfering with the de-ubiquitin modification of NLRP3 and directly targeting the NLRP3 protein upon bacterial infection. Because the inflammasome is increasingly being recognized as an important sensor and reactor to offending pathogens, our study showing how NleA interferes with this pathway provides important insights into efficient bacterial strategies for evading host immune responses and demonstrates the critical role of NLRP3 for the infection of A/E pathogens.

## Materials and Methods

### Cell culture and THP-1 differentiation

HeLa cells were maintained in MEM (Sigma) supplemented with 10% FCS and 1x NEAA (Gibco). RPMI-1640 medium containing 10% FCS and 1x NEAA was used for THP-1 culture. THP-1 cells were differentiated into adherent macrophage-like cells in a time course of 4 to 5 days before being used in experiments. On day 0, cells were first seeded at 2x10^5^ cell/ml in 24 well plates and stimulated with Phorbol 12-myristate 13-acetate (PMA) (Wako) at concentration of 100 ng/ml. On day 1, cells were washed with PBS once and fresh medium without PMA was added. Cells were then allowed to differentiate for the next 72–96 hrs. Half-medium change was carried out every 48 hrs.

### Bacterial strains, plasmids and oligonucleotides

The bacterial strains and plasmids used in this study are described in S1 Table. Primers used for cloning are listed in S2 Table. Construction of *nleA* deletion mutant, Δ*nleA*, followed published protocols [49]. Genes encoding effectors were PCR amplified directly from EHEC Sakai (Accession No. NC_002695) and E2348/69 (Accession No. FM180568) chromosomal DNAs. All generated DNA fragments were digested with designated restriction enzymes and subcloned into bacterial or mammalian expression plasmids as indicated.

### Bacterial infection

One day prior to the infection experiment, bacterial strains to be used were inoculated in selective LB broth overnight with constant agitation at 30°C. On the day of the experiment, bacterial cultures at the stationary phase were diluted 20-fold in serum-free DMEM (Sigma) and cultured with agitation at 37°C for 2 hours. Two hours before the actual infection, differentiated THP-1 was washed once with PBS and cell medium was changed to serum-free RPMI containing LPS at 1 μg/ml (Sigma). Upon completion of 2 hours of bacteria culture, bacteria were added to cell culture at m.o.i (multiplicity of infection) of 20 or as indicated and cells were subjected to 10 min of spin-infection at 1600 x rpm to synchronize the start of infection. Following 1 hr of incubation at 37°C, 5%CO_2_, gentamycin (Wako) was added at concentration of 0.1 mg/ml to terminate further infection by extracellular bacteria. Cells were further incubated and samples (cells or cell culture medium) were harvested for analysis at time indicated.

### ELISA assay

THP-1 were seeded at 2x10^5^ cells/ml in the 24 well plate and induced for differentiation as mention above. After the initial spin-infection and subsequent one hour of co-culturing, the live bacteria were terminated by addition of gentamycin. Cells were further cultured at 37°C, 5%CO_2_ for 6 hrs. The culture medium were collect and centrifuged once to pellet down bacterial debris as well as the non-adherent cells. Commercial ELISA kits (IL-1β and IL-8, both from eBioscience; caspase-1, R&D) were used to determine cytokine concentration in the medium, following the manufacture’s protocol.

### Immunofluorescent staining

THP-1 were seeded at 2x10^5^ cell/ml on the coverslips in the 24 well plate and induced for differentiation by PMA as described above. At the end of infection experiment, cells were fixed by 4% paraformaldehyde (PFA) and permeabilized with 0.1% Triton-X in PBS. Cells were blocked with 1%BSA in PBS and stained with primary antibodies overnight at 4°C, followed by treatment with appropriate Alexa Fluo-555 or Alexa-Fluo-488 secondary antibodies at room temperature for 1 hr (Invitrogen). Slides were mounted with *SlowFade* Gold antifade reagent with DAPI (Invirogen). For FLICA staining of active caspase-1, 1 hr prior to the fixation with 4% PFA, cells were washed with warm PBS to remove non-adherent bacteria and cell debris, and treated with FAM-YVAD-FMK peptides (immunochemistry) at concentration of 5 mM per manufacturer’s suggestion. Upon completion of one hour staining in 5%CO_2_ chamber at 37°C, cells were washed with PBS to remove non-binding reagents and fixed for subsequent immuno-staining procedure.

### Confocal microscopy and quantification of inflammasome structures

Samples were visualized under 60x oil immersion lens of Olympus FV10i-DOC. For each sample slide, 9 randomly selected fields of views were examined and image recorded with z-axis stacking mode. Raw images were exported by FLUOVIEW Viewer software (ver4.0, Olympus) in the format of TIFF. The exported files were processed by Adobe Photoshop CS5 (Adobe System). For quantification of active caspase-1, total caspase-1 and ASC foci, the number of cells contained at least one spherical or speckle-like structures were counted, divided by the number of cells per field, and expressed in percentage. For identification of mature NLRP3 foci, cells were co-stained with NLRP3 and caspase-1 specific antibody. At each indicated time point, cells that show co-localized signals in a speckle-like structure were counted. The percentage of foci formation was then calculated as the number of cells with foci divided by number of cells in the field, expressed in percentage.

### HeLa cell transfection

HeLa cells were seeded at 0.8x10^5^ cell/ml in the 24 well plate or 4x10^5^ cell/well in the 6 well plate or 3x10^6^ cell/10-cm dish one night prior to the transfection. On the day of transfection, cell culture were transfected using Lipofectamine 2000 per manufacture’s suggestion (Life Technologies). Cells were further cultured for indicated time before being collected for subsequent experiments.

### Immunoblotting and immunoprecipitation assay

For the whole cell lysate, cells were washed with PBS and directly lysed in 2x SDS sampler buffer and sonicated. For assessment of active caspase-1, collected cell culture medium were centrifuged at 800xg for 5 min to sediment cells and the supernatant were transferred to new eppendorf tubes for subsequent TCA precipitation. The precipitated proteins were dissolved in 1x SDS sampler buffer and were analyzed by immunoblotting.

Prepared samples were separated on SDS-PAGEs and procedures of immunoblotting were performed according to standard protocol. For Immunoprecipitation and co-immunoprecipitation assay, cells were washed and lysed in NP-40 lysis buffer (20 mM HEPES-KOH pH7.6, 150 mM NaCl, 1% NP40, 10% glycerol, 5 mM NaF and Proteinase inhibitor cocktail (Sigma)). Cells in the buffer were incubated on ice for 15 min, and centrifuged at 12,500xg for 15 min. The supernatant collected as the cytosolic fraction was added with species-matched control immunoglobulin G (IgG) or specific antibodies coupled to Dynabeads Protein G (Invitrogen) and allowed to mix at 4°C for overnight. The bound proteins were washed with lysis buffer and eluted in 1x SDS Sample buffer. For detection of NLRP3 ubiquitination, cells were lysed in RIPA buffer (50 mM Tris-HCl pH7.6, 150 mM NaCl, 1 mM EDTA, 0.05% sodium deoxycholate, 1% Triton X-100, 0.01% SDS, 10 mM *N*-ethylmaleimide). Endogenous NLRP3 proteins were immunopricipitated with anti-NRLP3 (Cell signaling technology) coupled to Dynabeads IgG (Invitrogen). After 3 hours of gentle agitation at 4°C, Dynabeads bound proteins were washed with RIPA buffer and eluted with 1x SDS sampler buffer.

### Direct binding assay

Bacterial expression plasmids containing each coding sequence were transformed into *E*.*coli* BL21(DE3) strain. Freshly transformed colonies were picked and cultured in selective broth LB one night before the induction. On the day of induction, overnight cultured bacteria was first diluted 500-fold in fresh LB and cultured with constant agitation at 37°C until O.D_600_ ~0.4. IPTG was then added to induce protein expression for the next 3 hours. Bacteria were collected and soluble proteins were extracted. Amylose resin and glutathione agarose beads were used to purify MBP-fusion and GST-fusion proteins, respectively, following manufacture’s suggestion (New England Biolabs, GE Life Sciences). To assess amount of bound proteins, known concentration of BSA standard and samples of purified proteins were separated on SDS-PAGEs and stained by CBB (Wako). To determine the direct binding between MBP-NleA and GST-NLRP3-FL (full length), equal amount of purified GST or GST-NLRP3-FL bound by the glutathione beads (total of 2.5 μg each) were first packed into columns at beads bed volume of 500 μl. Purified MBP-NleA at concentration of 5 μg/ml were applied to the column and allowed to flow-through by gravitation. Columns were washed with 0.1% Triton-X/PBS and eluted with high salt elution buffer (1x PBS supplemented with 850 mM NaCl). The eluted products were precipitated by TCA as described above. Concentrated samples were applied and separated on SDS-PAGE, followed by immunoblotting with anti-MBP antibody. Similar procedures were taken for determining NleA-interacting domains on NLRP3. For determination of interacting domains, same steps described above were taken.

### In vitro de-ubiquitination assay

Total KGC-NLRP3 was isolated by anti-KGC antibody (MBL) coupled magnetic beads from lysates of HeLa cells transfected with only the plasmids of KGC-NLRP3 or co-transfected with plasmids of KGC-NLRP3 and HA-ubiquitin. MBP and MBP-NleA fusion proteins were obtained as described in the section of Direct binding assay. To obtain LPS-primed dTHP-1 lysate, dTHP-1 was stimulated with LPS (1 μg/ml) for 2 hrs, lysed in assay buffer (25 mM Tris-Cl pH7.9, 100 mM NaCl, 1 mM DTT, 0.1% Triton X-100) and centrifuged at 15000xrpm at 4°C for 20 min. to obtain cytosolic fraction containing de-ubiquitin enzymes. For the de-ubiquitination assay, total KGC-NLRP3 bound on the magnetic beads were first mixed with MBP (2 μg) or MBP-NleA (2 μg) at 4°C for 16 hours. Then beads were washed twice with assay buffer to remove unbound proteins and re-suspended in 200 μl of assay buffer in new tube. Primed dTHP-1 lysates were added at 1/100 reaction volume. The de-ubiquitination reaction was allowed to proceed at 0°C or 37°C for 1 hr. At the end of reaction time, magnetic beads from each sample was washed three times with assay buffer and eluted in 1x SDS sampler buffer.

### Antibodies

Anti-caspase-1 (for immunoblotting; #2225, Cell Signaling), Anti-caspase-1 (for immunostaining; #3866, D7F10 clone, Cell Signaling), anti-ASC (D086-3, MBL), anti-GFP (AsOne), anti-Flag (M6, Sigma), anti-NF-κB (#4764, C22B4 clone, Cell Signaling), anti-IκBα (#4814, L35A5 clone, Cell Signaling), anti-NLRP3 (Cryo-2 clone, Adipogen), anti-NLRC4 (#12421, D5Y8E clone, Cell signaling), anti-ubiquitin (Ubi-1 clone, Millipore), anti-KGC (21B10 clone, MBL), anti-GFP mAb-Magnetic beads (RQ2 clone, MBL), and anti-Tubulin (Sigma) were used in this study.

### Statistical analysis and experiment reproducibility

The statistical analysis was calculated using the build-in mathematical function of Numbers ‘09 (Apple Inc.). Difference between two groups was identified using the Student’s *t*-test under the condition of two-tails/two-sample unequal for all analysis. Data are presented as means with standard error of mean (S.E.M) and statistical significance was recognized when p<0.05. All experiments were repeated for reproducibility and the representative data was shown in figures.

## Supporting Information

S1 Fig Amount of total associated and internalized bacteria per cell. THP-1 cells were infected by wild type, Δ*escF*, or TOE-A6 as described in Method. (A) Number of total associated bacteria were counted at 30 min after the end of infection, and (B) Number of internalized bacteria were counted at 1 hour after the end of infection with gentamicin treatment. Cell lysates were prepared by treatment of NP-40 lysis buffer. Lysates were diluted 1000-fold and plated on LB agar plates for overnight culture at 37°C. The number of associated or internalized bacteria was calculated by (number of colony) / (number of THP-1 used) • p < 0.05 (Student t-test).
(PDF)

S2 Fig NleE but not NleA inhibited pro-IL-1β production. Differentiated THP-1 was infected with indicated strains. 1.5 hours after the infection, cells were lysed and the cytosolic fraction was analyzed with an antibody specific to pro-IL-1β. α-tubulin serves as the loading control.
(PDF)

S3 Fig Cells retain sufficient ubiquitinated NLRP3 after either 15 min or 2 hrs of LPS stimulation with below detectable amount of caspase-1 activation. Prior to the experiment, differentiated THP-1 was washed with warm PBS to remove serum-containing medium. Fresh serum-free RPMI medium were then added to cell culture. Cells were left un-treated or treated with LPS at 1 μg/ml for indicated time before recovery. Ubiquitinated proteins were immunoprecipitated as described in the Method. Proteins in culture medium was TCA-precipitated. Proteins of each fraction were analyzed by immunoblotting. (CL = Cytosolic lysate; SN = Supernatant)
(PDF)

S4 Fig NleA reduced Caspase-1 activation by combination of pathogen and nigericin stimulation. Cells were primed with LPS (1 μg/ml) for 2 hrs and uninfected or infected with TOE-A5 or TOE-A5/*nleA* for 1.5 hr. Cells were thoroughly washed and stimulated with nigericin (1 μM) for 1 hr. The culture medium and cell lysates were analyzed by immunoblot using specific antibodies indicated.
(PDF)

S5 Fig Localization of bacterial delivered NleA-FLAG3 in the host cell. Differentiated THP-1 was infected with TOE-A5 (expressing FLAG3 from pFLAG3-CTC) or TOE-A5/*nleA* (expressing NleA-FLAG3 from pFLAG3-NleA) as described in Materials and Method. After 1 hr of infection, gentamicin was added to terminate the infection and cells were further incubated for 2 hrs. For immunofluorescent staining, cells were washed, fixed with 4% PFA and blocked with 5%BSA/PBS, followed by sequential staining with anti-FLAG and Alexa Fluo488 Goat anti-mouse-IgG. Slides were mounted with SlowFade/DAPI. Microscopic images were taken by the confocal microscope Olympus FV10i and were processed by Photoshop CC 2014.
(PDF)

S6 Fig Effect of NleA C-terminal deletion on Caspase1 activation and IL-1β secretion. Differentiated THP-1 were uninfected or infected with TOE-A5, TOE-A5 expression full length of NleA (TOE-A5/*nleA-FL*), deletion mutants lacking either C-terminus 33 or 62 amino acids (TOE-A5/*nleA-Δ33* or TOE-A5/*nleA-Δ62*, respectively). A. 3 hrs after the termination of infection, TCA-precipitated proteins from culture medium and cell lysates were analyzed by immunoblot using indicated antibodies, B. 6 hrs after the termination of infection, culture medium were collected and the amount of IL-1β was determined.
(PDF)

S1 Table Bacterial strains and plasmids. (PDF)

S2 Table Primers and oligonucleotides used in this study. (PDF)
