# Supplementary figures and images for: Enteropathogenic Escherichia coli Uses NleA to Inhibit NLRP3 Inflammasome Activation
Source: PLoS Pathog. 2015 Sep 2;11(9):e1005121. doi: 10.1371/journal.ppat.1005121 (PMC4557958; doi:10.1371/journal.ppat.1005121)

S1 Fig. Amount of Total associated and internalized bacteria per cell.

A

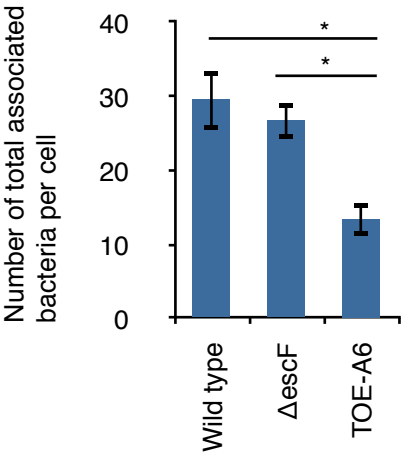

B

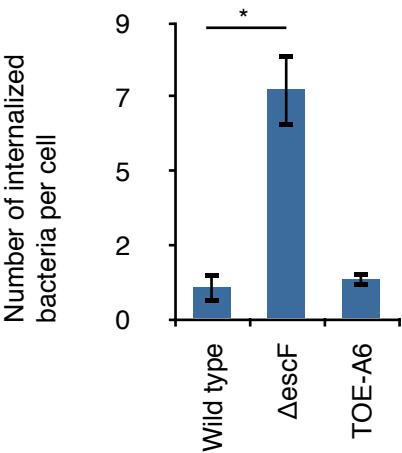

Supplement: S1 Fig — THP-1 cells were infected by wild type, ΔescF, or TOE-A6 as described in Method. (A) Number of total associated bacteria were counted at 30 min after the end of infection, and (B) Number of internalized bacteria were counted at 1 hour after the end of infection with gentamicin treatment. Cell lysates were prepared by treatment of NP-40 lysis buffer. Lysates were diluted 1000-fold and plated on LB agar plates for overnight culture at 37°C. The number of associated or internalized bacteria was calculated by (number of colony) / (number of THP-1 used) • p < 0.05 (Student t-test). (PDF) [file ppat.1005121.s001.pdf]

S2 Fig. NleE but not NleA inhibited Pro-IL-1 $\beta$  production

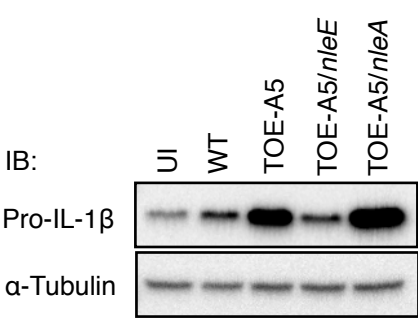

Supplement: S2 Fig — Differentiated THP-1 was infected with indicated strains. 1.5 hours after the infection, cells were lysed and the cytosolic fraction was analyzed with an antibody specific to pro-IL-1β. α-tubulin serves as the loading control. (PDF) [file ppat.1005121.s002.pdf]

**S4 Fig. NleA reduced Caspase-1 activation by combination of pathogen and Nigericin stimulation**

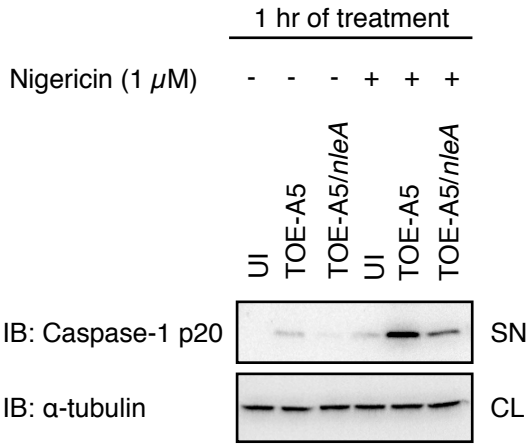

Supplement: S4 Fig — Cells were primed with LPS (1 μg/ml) for 2 hrs and uninfected or infected with TOE-A5 or TOE-A5/nleA for 1.5 hr. Cells were thoroughly washed and stimulated with nigericin (1 μM) for 1 hr. The culture medium and cell lysates were analyzed by immunoblot using specific antibodies indicated. (PDF) [file ppat.1005121.s004.pdf]

**S5 Fig. Localization of bacterial delivered NleA-Flag3 in the host cells.**

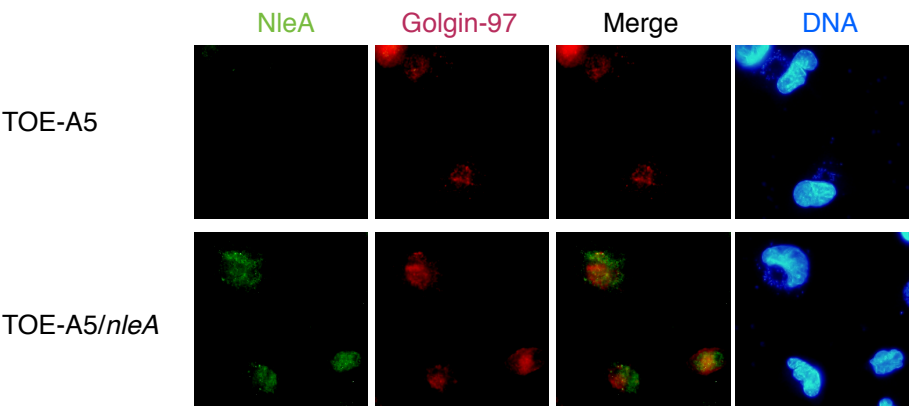

Supplement: S5 Fig — Differentiated THP-1 was infected with TOE-A5 (expressing FLAG3 from pFLAG3-CTC) or TOE-A5/nleA (expressing NleA-FLAG3 from pFLAG3-NleA) as described in Materials and Method. After 1 hr of infection, gentamicin was added to terminate the infection and cells were further incubated for 2 hrs. For immunofluorescent staining, cells were washed, fixed with 4% PFA and blocked with 5%BSA/PBS, followed by sequential staining with anti-FLAG and Alexa Fluo488 Goat anti-mouse-IgG. Slides were mounted with SlowFade/DAPI. Microscopic images were taken by the confocal microscope Olympus FV10i and were processed by Photoshop CC 2014. (PDF) [file ppat.1005121.s005.pdf]

S6 Fig. Effect of NleA C-terminal deletion on Caspase1 activation and IL-1 $\beta$  secretion.

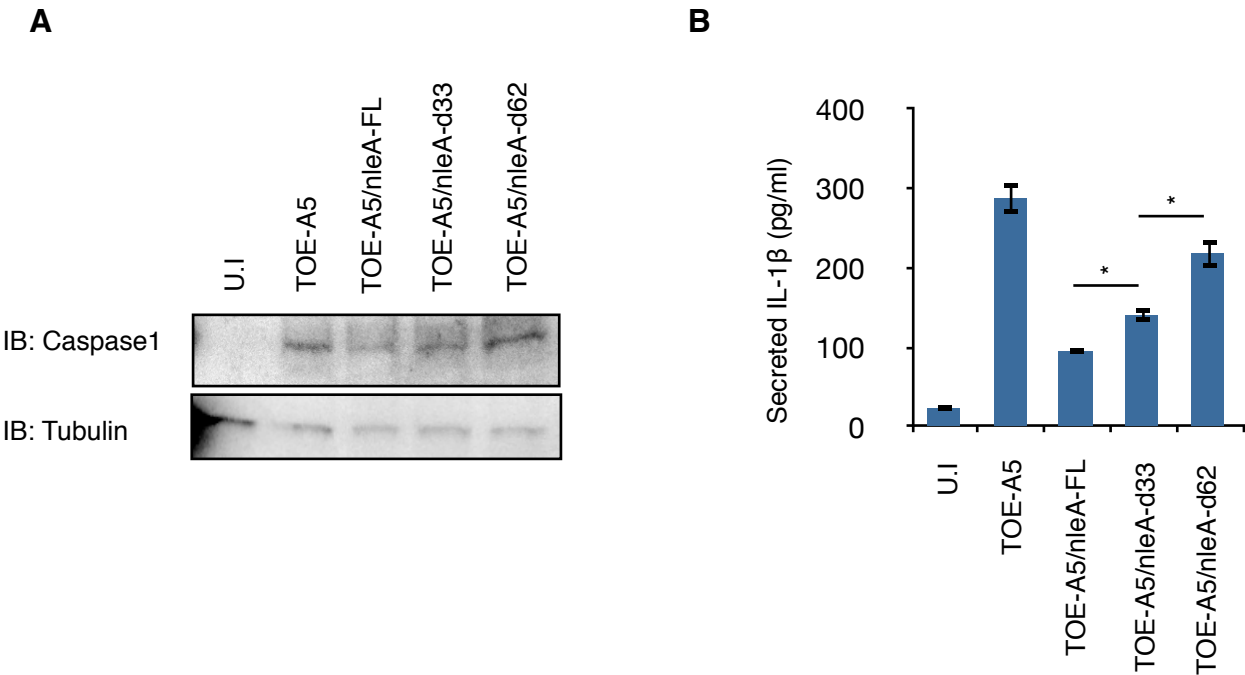

Supplement: S6 Fig — Differentiated THP-1 were uninfected or infected with TOE-A5, TOE-A5 expression full length of NleA (TOE-A5/nleA-FL), deletion mutants lacking either C-terminus 33 or 62 amino acids (TOE-A5/nleA-Δ33 or TOE-A5/nleA-Δ62, respectively). A. 3 hrs after the termination of infection, TCA-precipitated proteins from culture medium and cell lysates were analyzed by immunoblot using indicated antibodies, B. 6 hrs after the termination of infection, culture medium were collected and the amount of IL-1β was determined. (PDF) [file ppat.1005121.s006.pdf]
