# Supplementary material for: Enteropathogenic Escherichia coli Uses NleA to Inhibit NLRP3 Inflammasome Activation
Source: PLoS Pathog. 2015 Sep 2;11(9):e1005121. doi: 10.1371/journal.ppat.1005121 (PMC4557958; doi:10.1371/journal.ppat.1005121)

S3 Fig. Cells retain sufficient ubiquitinated NLRP3 after either 15 min or 2 hrs of LPS stimulation with below detectable amount of caspase-1 activation.

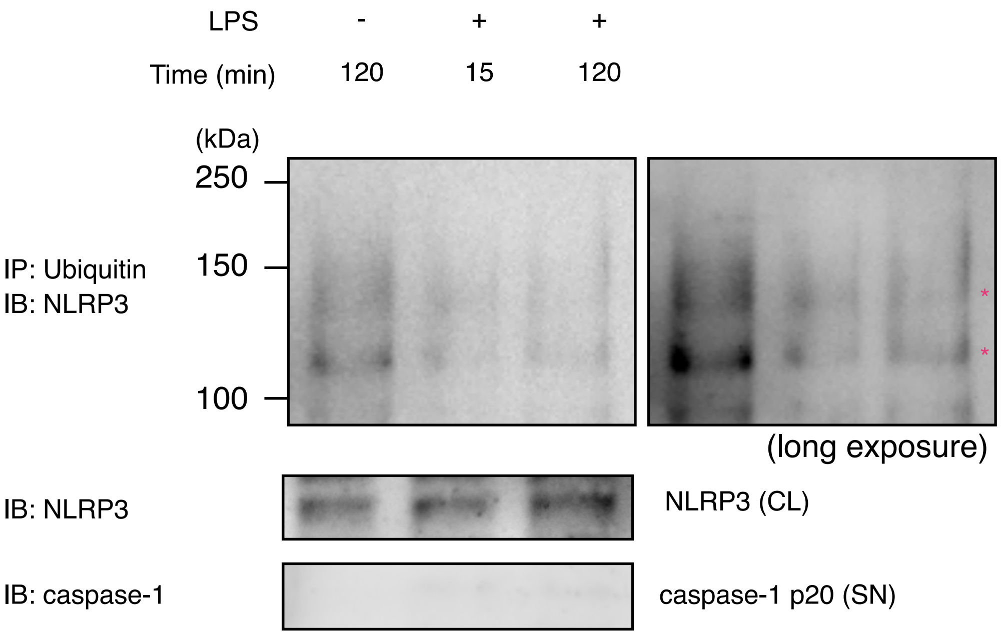

Supplement: S3 Fig — Prior to the experiment, differentiated THP-1 was washed with warm PBS to remove serum-containing medium. Fresh serum-free RPMI medium were then added to cell culture. Cells were left un-treated or treated with LPS at 1 μg/ml for indicated time before recovery. Ubiquitinated proteins were immunoprecipitated as described in the Method. Proteins in culture medium was TCA-precipitated. Proteins of each fraction were analyzed by immunoblotting. (CL = Cytosolic lysate; SN = Supernatant) (PDF) [file ppat.1005121.s003.pdf]
