# Supplementary material for: Enteropathogenic Escherichia coli Uses NleA to Inhibit NLRP3 Inflammasome Activation
Source: PLoS Pathog. 2015 Sep 2;11(9):e1005121. doi: 10.1371/journal.ppat.1005121 (PMC4557958; doi:10.1371/journal.ppat.1005121)
Supplement: S1 Table — (PDF) [file ppat.1005121.s007.pdf]

S1 Table Bacterial strains and plasmid

Strain names	Description	Reference
**EPEC E2348/69**	EPEC wild type	Iguchi et al. (2009)
**TB0661**	E2348/69 Δ*escF*	Hilo et al. (2010)
**TB2049**	E2348/69 Δ*nleA*	This study
**TOE-A1**	E2348/69-derivative: the *nleE/nleB/espL* cluster (on IE2)	Hilo et al. (2010)
**TOE-A2**	E2348/69-derivative: the *nleH/cif/espJ* cluster (on PP2)	Hilo et al. (2010)
**TOE-A3**	E2348/69-derivative: the *nleG/nleC/nleD* cluster (on IE2)	Hilo et al. (2010)
**TOE-A4**	E2348/69-derivative: the *nleE/nleB/espL* cluster (on IE6)	Hilo et al. (2010)
**TOE-A5**	E2348/69-derivative: the *nleH/nleA(espI)/nleF/espO* cluster (on PP6)	Hilo et al. (2010)
**TOE-A6**	E2348/69-derivative: the *espG2* (*orf3*) (on IE5) deleted	Hilo et al. (2010)
**Plasmid names**	**Description**	**Reference**
**pFLAG3-CTC**	FLAGx3 fusion plasmid: a derivative of pFLAG-CTC (Sigma)	Miyahara et al., Cellular Microbiology 2009
**pFlag3-NleE**	pFLAG3-CTC derivative: NleE-Flag3 expressing plasmid	This study
**pFlag3-NleB**	pFLAG3-CTC derivative: NleB-Flag3 expressing plasmid	This study
**pFlag3-EspL**	pFLAG3-CTC derivative: EspL-Flag3 expressing plasmid	Miyahara et al., Cellular Microbiology 2009
**pFlag3-NleA**	pFLAG3-CTC derivative: NleA-Flag3 expressing plasmid	This study
**pFlag3-NleF**	pFLAG3-CTC derivative: NleF-Flag3 expressing plasmid	This study
**pFlag3-NleH**	pFLAG3-CTC derivative: NleH2-Flag3 expressing plasmid	This study
**peGFP-C1**	mammalian expression plasmid for eGFP fusion protein	Clontech
**pF1KB3222**	pF1KOF derivative: NLRP3 clone	Kazusa DNA Res., Nagase et al., DNA Res 2008
**peGFP-NleA**	peGFP-C1 derivative: eGFP-NleA expressing plasmid	This study
**pKGC-MC**	mammalian expression plasmid for KGC fusion protein	MBL
**pKGC-MC-NLRP3**	pKGC-MC derivative: KGC-NLRP3 expressing plasmid	This study
**pcDNA3.1+mychis(A)**	mammalian expression plasmid for myc-his fusion protein	Invitrogen
**pcDNA3.1-HA-Ubi**	pcDNA3.1+mychis(A) derivative: HA-Ubiquitin expressing plasmid	This study
**pcDNA3-mRFP**	Obtained from Addgene. mRFP fusion plasmid. Plasmid#	Addgene
**pcDNA3-NLRP3-mRFP**	pcDNA3-mRFP derivative: NLRP3-mRFP expressing plasmid	This study
**pMAL-c2x**	Maltose-binding protein (MBP) fusion plasmid	New England Biolabs
**pMAL-NleA**	pMAL-c2x derivative: MBP-NleA expressing plasmid	This study
**pGEX-6P-1**	GST fusion plasmid	GE Healthcare Life Sciences
**pGEX-NLRP3**	pGEX-6P-1 derivative: GST-NLRP3 expressing plamid	This study
**pGEX-NLRP3-PYD**	pGEX-6P-1 derivative: GST-NLRP3-PYD expressing plamid	This study
**pGEX-NLRP3-NACHT**	pGEX-6P-1 derivative: GST-NLRP3-NACT expressing plamid	This study
**pGEX-NLRP3-LRR**	pGEX-6P-1 derivative: GST-NLRP3-LRR expressing plamid	This study

Reference:

Iguchi A, Thomson NR, Ogura Y, Saunders D, Ooka T, et al. (2009) J Bacteriol 191(1): 347-354

Akira Miyahara et al. (2009) Cellular Microbiology. 11(2): 337 - 350.

Hilo Y et al. (2010) PLoS Pathogen. 6(12): e1001231

Nagase, T., et al. (2008) DNA Res. 15:137-149
