# Supplementary material for: Enteropathogenic Escherichia coli Uses NleA to Inhibit NLRP3 Inflammasome Activation
Source: PLoS Pathog. 2015 Sep 2;11(9):e1005121. doi: 10.1371/journal.ppat.1005121 (PMC4557958; doi:10.1371/journal.ppat.1005121)
Supplement: S2 Table — (PDF) [file ppat.1005121.s008.pdf]

S2 Table Primers and oligonucleotides used in this study

Oligonucleotide	Sequence	Comment
**3857-N4**	GAAGGAATTCGATATTAACATGATTAGAACAAG	NleB
**3857-C4**	GTGAGTCGACTGAACTGCAGGTATACATACTGGT	NleB
**NleE-N-NdeI**	GTTGCATATGATTAATCCTGTTACTAATACTCAGG	NleE
**NleE-C-XhoI**	TGGTCTCGAGCTCAATTTTAGAAAGTTTATTATTTATGT	NleE
**1815-N2**	CTCTCATATGTTACCAACAAGTGGTTCTTC	NleF
**1815-C3**	GTGTCTCGAGTCCACATTGTAAAGATCCTTTGTTG	NleF
**1812-N2**	CATGCATATGAACATTCAACCGACCATAC	for pFLAG3-NleA
**NleA-R-XhoI-FL1**	CCTTCTCGAGGACTCTTGTTTCTTGGATTATATC	for pFLAG3-NleA
**ECs1814-N2**	CTACCATATGTTATCGCCCTCTTCTATA	NleH
**ECs1814-C4**	ATGGCTCGAGTTACTCCAGTCTGTCAAACATATCG	NleH
**BamH1-ATG-HA-Ubb-N**	cgcgGGATCCATGTACCCATACGACGTGCCAGATTACGCTATGCAGATCTTCGTGAAAACCCTT	HA-Ubiquitin
**EcoRI-TAA-Ubb-1-C**	cgcgGAATTCTTAACCACCTCTCAGACGCAGGACCAGG	HA-Ubiquitin
**NleA-F1-BglII**	GCATAGATCTGCCGCCACCATGAACATTCAACCGACCATACAATCTG	for peGFP-NleA
**NleA-R2-XhoI**	CCTTCTCGAGTTAGACTCTTGTTTCTTGGATTATATC	for peGFP-NleA
**NLRP3-F1**	CGATGGATCCGCCACCATGAAGATGGCAAGCACCCGC	for NLRP3 subcloning
**NLRP3-R1**	TGACCTCGAGCCAAGAAGGCTCAAAGACGAC	for pcDNA3-NLRP3-mRFP
**NLRP3-R2**	TGACCTCGAGTTACCAAGAAGGCTCAAAGACGAC	for pKGC-MC-NLRP3 and pGEX-6P-1-NLRP3
**NLRP3-213-R1**	TGACCTCGAGTTACACAGGCTCAGAATGCTCATC	for pGEX-6P-1-NLRP3-PYD
**NLRP3-213-F1**	CGATGGATCCGCCACCATGGATGAGCATTCTGAGCCTGTG	for pGEX-6P-1-NLRP3-NACHT
**NLRP3-398-R1**	TGACCTCGAGTTACAGACTGAAGGCTGCCCTGGC	for pGEX-6P-1-NLRP3-NACHT
**NLRP3-389-F1**	CGATGGATCCATGGCCAGGGCAGCCTTCAGTCTG	for pGEX-6P-1-NLRP3-LRR
